# Ultrasonographic image of a rare case of papillary thyroid carcinoma from a thyroglossal cyst

**DOI:** 10.11604/pamj.2025.52.79.49628

**Published:** 2025-10-20

**Authors:** Abdeljabbar Moussaoui, Faycal El Gendouz

**Affiliations:** 1 Department of Otolaryngology Head and Neck Surgery, Moulay Ismail Military Hospital, Meknes, Morocco,; 2 Department of Endocrinology and Metabolism, Moulay Ismail Military Hospital, Meknes, Morocco,; 3 Human Pathology, Biomedicine and Environment Laboratory, Faculty of Medicine, Pharmacy, and Dentistry of Fez, Sidi Mohammed Ben Abdellah University, Fez, Morocco

**Keywords:** Thyroglossal cyst, nodular formation, papillary thyroid carcinoma

## Image in medicine

A 28-year-old female patient with no significant medical history presented with a midline cervical mass. The lesion had been present since childhood but remained stable and asymptomatic until recently, when progressive enlargement prompted medical consultation. Clinical examination revealed a firm, mobile, non-tender midline cervical swelling with low mobility during swallowing. Overlying skin appeared normal without inflammatory signs. The remainder of the otolaryngological examination was unremarkable. Cervical ultrasonography identified a complex septated cystic nodular formation containing a vascularized isoechoic solid component, the thyroid was multinodular with no indication for fine needle biopsy and there were no lymphadenopathies. The patient underwent complete surgical excision via the Sistrunk procedure. Histopathological examination of the resected specimen unexpectedly revealed papillary thyroid carcinoma arising within a thyroglossal duct cyst. Additional treatment by total thyroidectomy was discussed with the respiratory care practitioner (RCP) in the presence of a multinodular thyroid. This case illustrates an unusual presentation of thyroid carcinoma within a thyroglossal duct cyst, highlighting the importance of comprehensive evaluation of even classic-appearing congenital cervical lesions. The ultrasonographic identification of a solid, vascularized component within a suspected thyroglossal duct cyst should raise suspicion for malignant transformation and guide appropriate surgical management.

**Figure 1:**
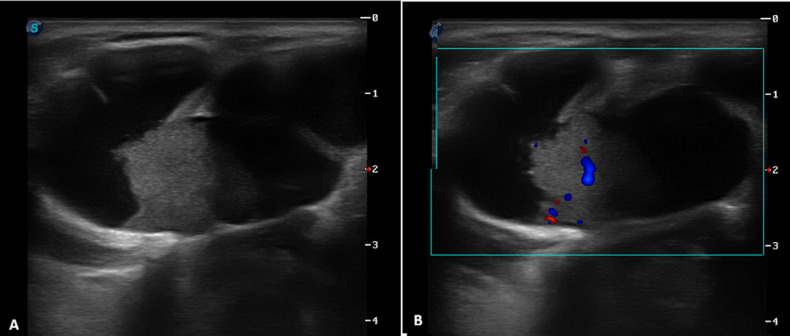
cross-section of cervical ultrasound showing a septated midline cyst located above the thyroid cartilage, measuring 28x50x30 mm; B) an isoechoic tissue component adherent to the posterior cyst wall, measuring 12x11x10 mm, with central vascularization

